# Structural and Functional Characteristics of Mitral Paravalvular Leakage Identified by Multimodal Imaging and Their Implication on Clinical Presentation

**DOI:** 10.3390/jcm10020222

**Published:** 2021-01-10

**Authors:** Jah Yeon Choi, Young Joo Suh, Jiwon Seo, Kang-Un Choi, Geu-Ru Hong, Sak Lee, Seung-Hyun Lee, Jong-Won Ha, Young Jin Kim, Chi Young Shim

**Affiliations:** 1 Cardiovascular Center, Korea University Guro Hospital, Korea University College of Medicine, Seoul 08308, Korea; kekeruki@gmail.com; 2 Department of Radiology, Research Institute of Radiological Science, Severance Hospital, Yonsei University College of Medicine, Seoul 03722, Korea; RONGZU@yuhs.ac; 3 Division of Cardiology, Department of Internal Medicine, Severance Cardiovascular Hospital, Yonsei University College of Medicine, Seoul 03722, Korea; EPRANGIANA@yuhs.ac (J.S.); GRHONG@yuhs.ac (G.-R.H.); JWHA@yuhs.ac (J.-W.H.); 4 Division of Cardiology, Department of Internal Medicine, Donguk University Gyeongju Hospital, Gyeongju 38067, Korea; tipcode@gmail.com; 5 Department of Cardiothoracic Surgery, Severance Cardiovascular Hospital, Yonsei University College of Medicine, Seoul 03722, Korea; SAK911@yuhs.ac (S.L.); HENRY75@yuhs.ac (S.-H.L.)

**Keywords:** mitral paravalvular leakage, hemolysis, pulmonary hypertension, heart failure, multimodal imaging

## Abstract

Objective: Clinical presentation of patients with mitral paravalvular leakage (PVL) varies from asymptomatic to heart failure related with hemolytic anemia or pulmonary hypertension. We aimed to investigate the structural and functional characteristics of mitral PVL by multimodal imaging and their association with the severity of hemolysis and hemodynamic significance. Methods: A total of 74 patients with mitral PVL who underwent both cardiac computed tomography (CT) and echocardiography from March 2010 to December 2017 was investigated. Location and size of PVL, degree of left atrial (LA) calcification as measured by CT, and hemodynamic variables as measured by echocardiography were comprehensively analyzed. To investigate the degree of hemolysis and pulmonary hypertension, level of lactate dehydrogenase (LDH) and Doppler estimated systolic pulmonary artery pressure (SPAP) were used respectively. Results: Level of LDH was not related to PVL perimeter and was variable, especially in patients with a small PVL. However, it was positively correlated with mean mitral regurgitation velocity. Additionally, SPAP was significantly correlated with PVL perimeter and LA calcium score. In multivariable analysis, mean mitral regurgitation velocity was significantly correlated with levels of LDH (β = 0.345; *p* = 0.016), and PVL perimeter and LA calcium score were independently associated with SPAP (β = 0.249; *p* = 0.036 and β = 0.467; *p* < 0.001, respectively). Conclusions: Characteristics of mitral PVL and adjacent structures are associated with the severity of hemolysis and pulmonary hypertension. Evaluating the structural and functional characteristics of mitral PVL by complementary multimodal imaging would be important for understanding the clinical presentation and deciding optimal treatments for individual patients.

## 1. Introduction

Paravalvular leakage (PVL) after mitral valve (MV) surgery is not uncommon, with rates varying from 7% to 17% after surgical implantation of prosthetic valves or annuloplasty rings [1]. Patients with PVL often have diverse symptoms and signs ranging from asymptomatic to hemolytic anemia or heart failure [1,2]. Despite recent advances in surgery and intervention, patients with PVL have poor clinical outcome due to high surgical mortality or incomplete percutaneous closure of the PVL [3,4,5]. Echocardiography is commonly used during an initial diagnosis of PVL. However, limitations in echocardiography exist due to acoustic shadowing caused by a prosthetic valve and a lack of information regarding the surrounding structural characteristics of the prosthesis. Recently, cardiac computed tomography (CT) has been used in patients with complicated heart valve disease, which can provide structural information regarding either the location of mitral PVL or specific pathologies such as endocarditis [6,7,8,9]. Multimodal imaging is thought to play a critical role in patients with mitral PVL; however, available data that explain the structural and functional characteristics of PVL and its clinical relevance are scarce.

In this study, we aimed to investigate (1) the structural and functional characteristics of mitral PVL and nearby structures by echocardiography and cardiac CT and (2) their associations with hemolysis and pulmonary hypertension, which can possibly result in heart failure.

## 2. Methods

### 2.1. Study Population

We retrospectively probed the clinical data for patients with prior MV surgery, repair, or replacement who underwent both cardiac CT and echocardiography between March 2010 and December 2017. Excluding 43 patients that had a time interval between CT and echocardiography that was greater than 90 days, and 36 patients without multiphase CT data, a total of 327 patients was included in our study. After excluding another 253 patients without PVL confirmed by CT and echocardiography, we finally enrolled 74 patients diagnosed with mitral PVL. In the 74 patients with PVL, cardiac CT was usually performed by clinician’s discretion when prosthetic valve dysfunction was suspected. Demographic data and information on surgical findings were collected from electronic medical records. The institutional review board of our institution approved this retrospective study, and informed consent was waived.

### 2.2. Echocardiography

All subjects underwent standard two-dimensional transthoracic echocardiography (TTE) and/or transesophageal echocardiography (TEE) within 90 days from CT. Conventional two-dimensional and Doppler measurements were performed as recommended by the American Society of Echocardiography guidelines using commercially available equipment [10]. The calculated systolic pulmonary artery pressure (SPAP) was defined as [4 × (maximum velocity of tricuspid regurgitant jet)^2^ + right atrial pressure]. Right atrial pressure was estimated by measuring inferior vena cava diameter and its response to inspiration. A comprehensive evaluation was performed to evaluate prosthetic or repaired MV function using routine and modified views. Valvular function was evaluated using Doppler measurements, such as peak or mean diastolic pressure gradients for prosthetic or repaired MV. A high-velocity, eccentric turbulent jet, with its origin beyond the edge of the sewing ring, was considered to indicate PVL [7,11]. The severity of PVL was evaluated using maximal widths of the vena contracta, and mild, moderate, and large PVL were defined based on width less than 3 mm, 3 to 6 mm, and 7 mm or more, respectively [11,12]. Mitral regurgitation (MR) velocity through PVL was measured using continuous-wave Doppler in standard view or off-axis view of TTE that can show MR contour most clearly (Supplemental Figure S1). TEE was performed at the clinician’s discretion using a Philips iE33 ultrasound system and an X7-2t transesophageal transducer (Philips Medical Systems, Andover, MA, USA).

### 2.3. Cardiac CT Acquisition

All CT scans were performed with a dual-source CT scanner (SOMATOM Definition Flash; Siemens Healthcare, Forchheim, Germany) or a wide coverage, 256-detector row CT scanner (Revolution CT; GE Healthcare, Waukesha, WI, USA) with an electrocardiographically gated data-acquisition mode. CT scans were performed with the triple-phase injection method (70 mL of iopamidol followed by 30 mL of 50% blended iopamidol with saline solution and 20 mL of saline solution at 5 mL/s).

From raw data sets, image reconstruction was performed with a medium kernel and iterative reconstruction, where the reconstruction slice thickness was 0.625 to 0.75 mm with 0.5- to 0.625-mm increments between slices. For all patients, 10 transverse datasets were reconstructed for every 10% of the cardiac cycle (0–90%), and reconstructed images were transferred to an image server and analyzed with dedicated three-dimensional (3D) software (Aquarius iNtuition, version 4.4.11; TeraRecon, Inc., San Mateo, CA, USA).

### 2.4. Cardiac CT Analysis

A dedicated radiologist who was blinded to clinical information and the results of TTE and TEE performed all the CT analyses. Assessment of the mitral PVL was performed using multiplanar reformatted images in cine mode. The short-axis view of the MV was used to assess the presence and location of the PVL. If PVL was present, the location was recorded using a localization system based on the surgical view and classified as anterolateral, anteromedial, posteromedial, or posterolateral. Mitral PVL size was assessed by measuring the area and perimeter on the short-axis view. The size of the mitral PVL was assessed in mid-systolic phase (in the first 20% to 30% of the R–R interval) by measuring the area and perimeter on the short-axis view. These measurements were confirmed to be concordant between two radiologists in a previous study by our group [7].

To add structural information from around the prosthetic MV, the left atrial (LA) calcium score was measured on a noncontrast-enhanced electrocardiography-gated scan. After identifying LA calcification greater than 130 Hounsfield units, the degree of LA calcification was quantified using the Agatston score system. Visual grading of LA calcification was performed according to extent of calcification within LA circumference and classified into four groups based on the corresponding score as follows: no LA calcification (0), mild LA calcification (1–500), moderate LA calcification (501–5000), and severe LA calcification (>5000).

### 2.5. Clinical Presentation

Presence of hemolytic anemia was defined as hemoglobin level <13 g/dL in women and <15 g/dL in men with one or more laboratory signs of hemolysis (i.e., increased bilirubin, lactic dehydrogenase (LDH), haptoglobin, or reticulocytosis abnormalities in peripheral blood smears) [13,14]. Level of LDH by blood chemistry was used to estimate the degree of hemolysis. Heart failure was defined as symptoms of dyspnea, signs of pulmonary congestion on chest X-ray, or increased level of N-terminal pro b-type natriuretic peptide (NT-proBNP) at the time of CT or echocardiographic imaging [15,16]. Noninvasively estimated SPAP by Doppler methods was used as an indicator of pulmonary hypertension.

### 2.6. Statistical Analysis

Continuous variables are presented as mean ± standard deviation, while categorical variables are expressed as percentages. The patient groups were compared using Student’s *t*-test for continuous variables and *χ*^2^ statistics for categorical variables. Mean values of LDH between groups of four different PVL locations were compared by analysis of variance (ANOVA). Simple linear regression analysis was performed to determine the associations between PVL parameters and the degree of hemolysis and heart failure. The variables that showed *p* < 0.1 during univariate analysis as well as age and sex were entered into a multivariable linear regression model and described with standardized β- and *p*-values. A value of *p* < 0.05 was considered significant. All statistical analyses were performed using the Statistical Package for the Social Sciences version 22.0 software program (IBM Corp., Armonk, NY, USA).

## 3. Results

### 3.1. Baseline Characteristics

The baseline clinical characteristics and data of valve surgery are presented in Table 1. The mean age of the study subjects was 63 years, and 31 patients (41.9%) were male. The majority of patients underwent MV replacement with prosthetic mechanical valve placement. More than one-third of the patients had already undergone cardiac surgery more than two times. Fifty patients (70.3%) had symptoms of heart failure, and the average NT-pro BNP was 1465 ± 1799 pg/mL. Additionally, the averages of hemoglobin and LDH were 8.4 ± 1.8 g/dL and 1450 ± 1022 IU/L, respectively. Thirty-one patients (41.9%) required blood transfusions.

### 3.2. Echocardiographic and Cardiac CT Characteristics

Table 2 presents echocardiographic and cardiac CT characteristics of the patients. The feasibility of CT imaging analysis was 89% (66/74 cases); there were eight cases showed suboptimal CT image quality due to motion or metal artifacts. Regarding the echocardiographic data, the averages of left ventricular (LV) dimension and ejection fraction were within normal ranges. Average LA volume index was 128.6 ± 99.1 mL/m^2^, and mean diastolic pressure gradient through the MV was 6.4 ± 2.5 mmHg. In terms of PVL severity, proportions of mild, moderate, and severe PVL were 54.1%, 32.4%, and 13.5%, respectively. Average peak MR velocity through the PVL was 5.4 ± 0.9 m/s, and mean MR velocity was 3.7 ± 0.7 m/s. The estimated SPAP was 55.2 ± 15.2 mmHg.

Additionally, various characteristics of PVL and LA were assessed by cardiac CT. Mean hole area and perimeter of PVL were 0.83 ± 0.87 cm^2^ and 41.3 ± 24.8 mm, respectively. The most common PVL location was the posterolateral, followed by posteromedial, anteromedial, and anterolateral. Patients without LA calcification composed only 27.0% in this population. The prevalence rates of mild, moderate, and severe LA calcification were 33.8%, 23.0%, and 13.5%, respectively. The average LA calcium score was 3507 ± 8057. Intriguingly, there was no difference in hole area and perimeter of PVL in three groups, which were divided into mild, moderate, and severe PVL according to the current guideline (Supplemental Figure S2).

### 3.3. Factors Associated with Hemolysis

**Figure 1:**
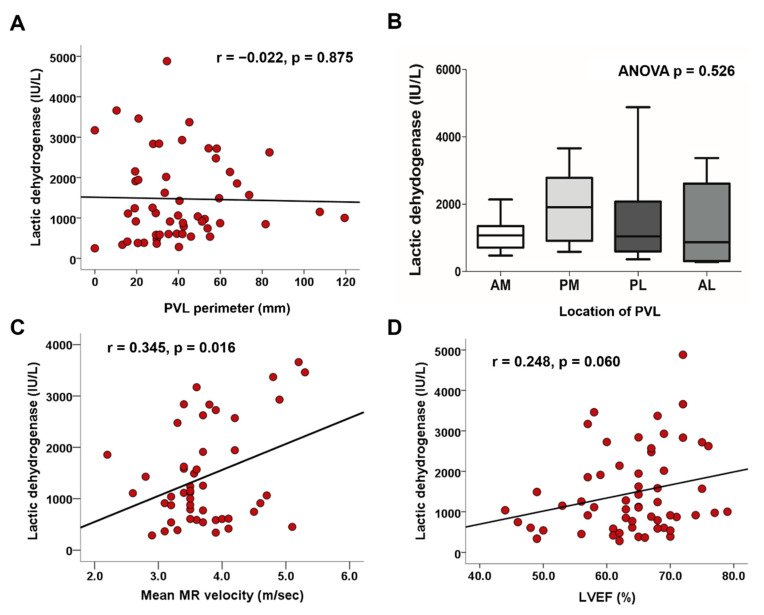
Associations between degree of hemolysis and (**A**) PVL perimeter, (**B**) mean MR velocity through the PVL, (**C**) PVL location, and (**D**) left ventricular ejection fraction. MR, mitral regurgitation; PVL, paravalvular leakage.

**Figure 2:**
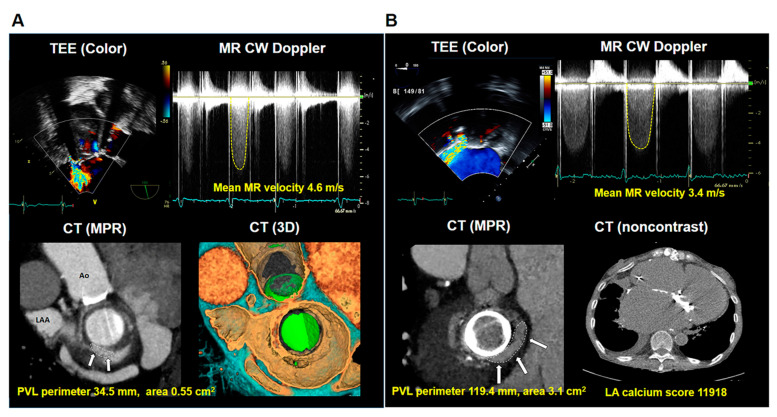
(**A**) Mitral PVL in a patient who presented with hemolytic anemia (hemoglobin: 7.4 g/dL and LDH: 4880 IU/L). Echocardiography demonstrates PVL at a posterolateral location with mean MR velocity of 4.6 m/s on continuous-wave Doppler. Multiplanar reconstruction and three-dimensional volume-rendering CT images from the surgeon’s view demonstrated the size of the PVL (white arrow). (**B**) Mitral PVL in a patient who presented with heart failure (pulmonary congestion with SPAP: 57 mmHg, hemoglobin: 9.4 g/dL, and LDH: 1001 IU/L). Echocardiography demonstrates PVL at an anteromedial location with a mean MR velocity of 3.4 m/s on continuous-wave Doppler. Multiplanar reconstruction CT demonstrated the large size of the PVL (white arrow), and noncontrast CT revealed LA calcification. Ao, aorta; CT, computed tomography; CW, continuous wave; LA, left atrium; LAA, left atrial appendage; LDH, lactate dehydrogenase; MPR, multiplanar reconstruction; MR, mitral regurgitation; 3D, three-dimension; PVL, paravalvular leakage; SPAP, systolic pulmonary artery pressure; TEE, transesophageal echocardiography.

Using a simple correlation analysis, PVL perimeter was not significantly correlated with levels of LDH (r = −0.022; *p* = 0.875) (Figure 1A). Similarly, PVL hole area on CT scans was not significantly correlated with levels of LDH (r = 0.036; *p* = 0.794). Notably, levels of LDH were highly variable when PVL hole area was smaller than 1.0 cm^2^ (Supplemental Figure S3). Regarding location of PVL, LDH levels were not different between the four groups (Figure 1B). There was a significant positive correlation between levels of LDH and mean MR velocity (r = 0.345; *p* = 0.016) (Figure 1C), however not peak MR velocity (r = 0.271; *p* = 0.062); which was consistent in the analysis of TEE (Supplemental Figure S4). There was no significant correlation between MR velocity and PVL size (Supplemental Figure S5). LV ejection fraction showed a positive correlation with levels of LDH; however, it was not significant (r = 0.248; *p* = 0.060) (Figure 1D). In multivariable linear regression, levels of LDH displayed a significant positive correlation with mean MR velocity (β = 0.345; *p* = 0.016) (Table 3). A representative case of mitral PVL presenting with hemolytic anemia is detailed in Figure 2A.

### 3.4. Factors Associated with Pulmonary Hypertension

**Figure 3:**
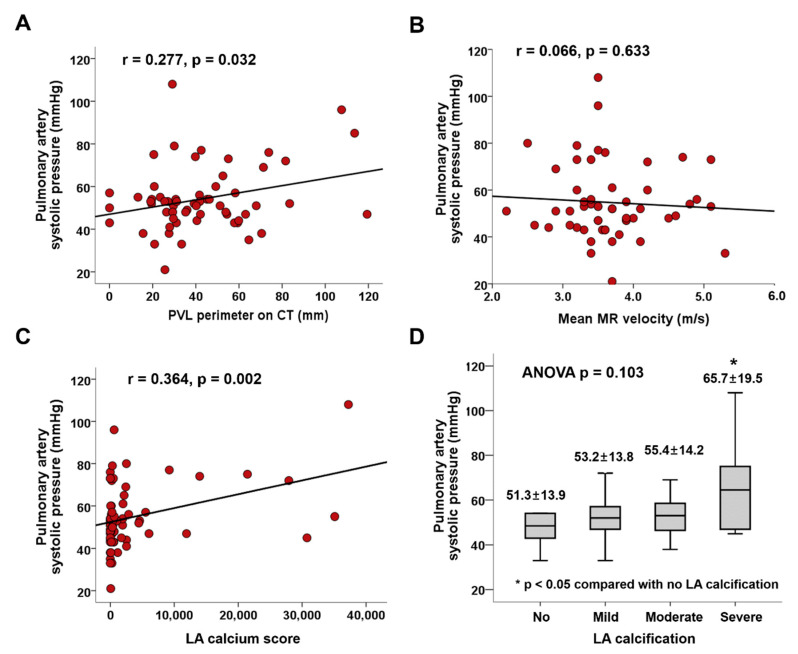
Associations between SPAP and (**A**) PVL perimeter, (**B**) mean MR velocity through the PVL, (**C**) LA calcium score, and (**D**) severity of LA calcification. LA, left atrium; MR, mitral regurgitation; PVL, paravalvular leakage; SPAP, systolic pulmonary artery pressure.

Larger PVL hole size, perimeter, and area were associated with significantly higher SPAP (r = 0.277; *p* = 0.032 and r = 0.272; *p* = 0.037, respectively) (Figure 3A). There was no association between SPAP and MR jet velocity (Figure 3B). Intriguingly, we found that a high LA calcium score correlated with high SPAP (Figure 3C), and SPAP was significantly higher in patients with severe LA calcification compared with those without LA calcification (65.7 ± 19.5 vs. 51.3 ± 13.9 mmHg; *p* = 0.045) (Figure 3D). In multivariable linear regression, SPAP showed significant positive correlations with LA calcium scores and PVL perimeters (β = 0.467; *p* < 0.001 and β = 0.249; *p* = 0.036, respectively) (Table 4). A representative case of mitral PVL presenting with heart failure is demonstrated in Figure 2B.

## 4. Discussion

**Figure 4:**
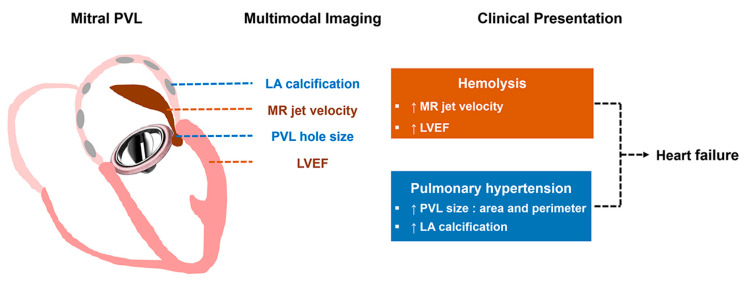
Structural and functional characteristics of mitral PVL determined by multimodal imaging and their clinical significance. PVL, paravalvular leakage.

The principal findings of the present study are as follows: (1) degree of hemolysis in mitral PVL was not related to PVL size and was particularly variable in patients with a small PVL; (2) degree of hemolysis was closely associated with mean MR velocity; and (3) The hemodynamic index of pulmonary hypertension was significantly associated with PVL size and severity of LA calcification. From the study findings, we suggest a complementary role for multimodal imaging in the evaluation of structural and functional characteristics of mitral PVL and adjacent chambers (Figure 4).

PVL is defined as abnormal communication between the sewing ring of a surgical prosthesis and the native annulus [11,12]. Several risk factors have been associated with the development of PVL such as surgical technique, type of prosthesis, and comorbidities such as mitral annular calcification or endocarditis [1]. Although surgery is indicated in symptomatic patients with conditions such as heart failure and hemolytic anemia [9,17], this subpopulation showed increased morbidity and mortality due to high surgical risk [18]. Recently, percutaneous closure of PVL has emerged as a possible alternative treatment strategy with high surgical risk [9,17]. Successful percutaneous intervention resulted in decreased cardiac-related mortality and symptoms of heart failure or hemolysis [19,20], and better clinical outcomes compared to surgery [21,22]. However, heart failure or hemolytic anemia might not improve after PVL closure if residual regurgitation is substantial [23,24]. To improve the clinical outcomes in patients with PVL, the professional experience and training with increased procedural volume would be indispensable. Furthermore, there needs to comprehensively evaluate the various characteristics of PVLs and its clinical implications so that physicians can find appropriate indications for treatment strategies.

A variety of diagnostic tests should be performed to evaluate PVL. Echocardiography is the initial choice of imaging modality for PVL diagnosis and offers valuable information related to hemodynamic consequences of PVL, such as valve function, heart chamber size and function, and pulmonary artery pressure. However, grading and quantification of PVL using conventional echocardiographic parameters, such as vena contracta, or the effective regurgitant orifice area and regurgitant volume measured by the proximal isovelocity surface area method, is a considerable challenge because PVL is usually observed as eccentric regurgitation. In our study, there was no significant difference in PVL hole area and perimeter on cardiac CT between three groups of PVL severity according to the current guideline. Transesophageal echocardiography with 3D reconstruction can provide more precise information regarding the location of PVL and is a crucial imaging modality for intraprocedural guidance. Some recent studies using multiplanar reconstruction of 3D TEE imaging reported acceptable accuracy in the quantitative measurement of PVL size on 3D color Doppler image [25,26].

Cardiac CT demonstrates excellent spatial resolution and can provide data on the aforementioned structural characteristics of PVL, which is helpful when planning and providing guidance for treatment [27]. Recently, the Valve Academic Research Consortium recommended that CT angiography be performed prior to reoperation for PVL [28]. Multimodal imaging thus has a complementary role in comprehensive evaluation of PVL in that structural and functional characteristics of PVL can be thoroughly evaluated with cardiac CT and echocardiography. 4D flow MRI, another novel imaging could be a complementary imaging modality that could assess both structural and hemodynamic aspects of heart; however, further study would be needed to prove its utility in PVL [27].

In the current study, using cardiac CT and echocardiography, we demonstrated that the degree of hemolysis was closely associated with mean MR velocity, and that degree of pulmonary hypertension was closely related to size of PVL and surrounding structures such as LA calcification.

The size of PVL was not associated with the degree of hemolysis, but functional factors such as MR velocity through the PVL and LV ejection fraction were related to degree of hemolysis. Hemolytic anemia results from shear stress on red blood cells. Besides, the interaction of blood with a foreign prosthetic material, and higher velocity through the PVL may facilitate a higher degree of hemolysis [29]. A previous study demonstrated that patients with mitral PVL were more symptomatic and presented with persistent hemolytic anemia and worse clinical outcomes relative to those with aortic PVL [2]. This may be due to the higher velocity and pressure gradient associated with mitral PVL because regurgitation occurs at the systolic phase. In contrast, regurgitation occurred at the diastolic phase in the aortic PVL and showed lower velocity and pressure gradient [2]. The results of our study agree with the previous findings [2]. We found that there was no significant correlation between PVL hole size and mean MR velocity through PVL. MR velocity through PVL can be determined not only by PVL size but also by various characteristics of PVL and surrounding structure such as pressure or compliance of LA and LV, high output state of anemia, and the degree of flow through PVL. We assume that LV systolic function, which showed a statistically insignificant correlation with the level of LDH but showed a marginal positive correlation, is one of the variables that can affect MR velocity. In our study, mean MR velocity was significantly associated with elevated LDH, however, peak MR velocity was not. The statistical insignificance of peak MR velocity may imply that the average shear stress through PVL would be more representable for demonstrating the burden of hemolysis than the peak shear stress.

The degree of pulmonary hypertension was closely related to PVL hole area and perimeter, which are structural characteristics of PVL. Recently, several studies reported additional value of CT in the evaluation of PVL [30,31]. These studies hypothesized an adjunctive role for CT; however, as they only involved 13 or 26 cases of mitral PVL, the results were not conclusive. In our study, we enrolled 74 patients with mitral PVL who underwent comprehensive evaluation of PVL and from whom detailed data were collected regarding hemolysis and heart failure. Furthermore, to evaluate the structural substrate affecting LA compliance, we investigated LA calcification and quantified the LA calcium score using the Agatston scoring system. Massive LA calcification, termed “porcelain atrium,” is considered to represent markedly decreased LA compliance and may cause elevated LA pressure and heart failure [32,33]. Recent study including patients with previous MV surgery demonstrated that increased SPAP in patients with severe LA calcification, and the level of SPAP was higher in the patients with PVL [34].

### 4.1. Study Limitations

First, this study was retrospective and cross-sectional in design, which has inherent potential limitations. We only count heart failure or hemolytic anemia episodes occurring at the time of CT and echocardiography imaging, not later during the follow-up period, and compared their association with various parameters on multimodal imaging. And, as cardiac CT was usually performed by clinician’s discretion when prosthetic valve dysfunction was suspected, there would be a relatively small number of patients without symptom or patients with renal insufficiency in our study population which resulted in selection bias. Second, although pulmonary hypertension can be induced or aggravated by hemodynamic consequence of mitral PVL, the chronic pulmonary artery remodeling caused by preoperative MV pathologies may affect the level of SPAP. In addition, we did not assess SPAP and other hemodynamic parameters by cardiac catheterization in this study. As for the hemolysis, there has been no single parameter recommended for evaluating the degree of hemolysis and each parameter of hemolysis, as each parameter can be affected by various clinical conditions. The level of LDH is thought to be a reliable marker for quantifying red cell damage in patients with valve replacement [35], and it was followed up to assess the degree of hemolysis after transcatheter PVL closure in previous studies [35,36]. Third, there were eight cases that showed suboptimal CT image quality and could not be evaluated due to motion or metal artifacts, and not all the patients underwent TEE for the evaluation of mitral PVL. Nevertheless, as far as we know, we enrolled the largest number of mitral PVL subsets and reported on various structural and functional characteristics of PVL by echocardiography and cardiac CT and their association with clinical presentation of PVL for the first time. Our results are expected to be helpful in understanding the hemodynamics of PVL and clinical consequences of the condition.

### 4.2. Clinical Implications

Patients with mitral PVL may present with hemolytic anemia and pulmonary hypertension, which can result in clinical heart failure. In this analysis, characteristics of mitral PVL and adjacent structures were associated with the severity of hemolysis and pulmonary hypertension. The degree of hemolysis was related to the mean mitral regurgitation velocity rather than the size of PVL, and the hemodynamic index of pulmonary hypertension was significantly related to PVL size and severity of LA calcification. From these results, we would like to highlight the complementary role of multimodal imaging in the evaluation of mitral PVL for understanding its clinical presentations and treatment response. Considering high mortality and morbidity in patients with PVL [22], we believe that comprehensive evaluation of mitral PVL may help physicians determine treatment strategy from patient selection for surgery or intervention to prediction of prognosis. Further studies on structural and functional changes before and after PVL closure as well as improvement of clinical presentations and outcomes should be conducted.

## 5. Conclusions

Cardiac CT has an advantage in evaluating structural characteristics of mitral PVL, and surrounding structures such as LA calcification whereas echocardiography is beneficial in assessing functional and hemodynamic characteristics related to the mitral PVL. The imaging modalities have a complementary role in evaluation of mitral PVL and are crucial for understanding the clinical presentation of patients with mitral PVL. Understanding the characteristics of mitral PVL with multimodal imaging is important to improve clinical outcome.

## Figures and Tables

**Table 1:** Baseline characteristics.

Variables	*n* = 74
Age, year	62.9 ± 9.0
Male, *n* (%)	31 (41.9)
Hypertension, *n* (%)	42 (56.8)
Diabetes mellitus, *n* (%)	18 (24.3)
Dyslipidemia, *n* (%)	23 (31.1)
Chronic kidney disease, *n* (%)	13 (17.6)
End-stage renal disease, *n* (%)	4 (5.4)
Atrial fibrillation, *n* (%)	62 (83.8)
Warfarin use, *n* (%)	74 (100.0)
Statin use, *n* (%)	26 (35.1)
Type of MV surgery, *n* (%)	
MV repair	1 (1.4)
MV replacement, bioprosthetic valve	6 (8.1)
MV replacement, mechanical valve	67 (90.5)
Number of MV surgery, *n* (%)	
Once	47 (63.5)
Twice	24 (32.4)
Three times or more	3 (4.1)
Time to last MV surgery to diagnosis of PVL, year	16.6 ± 8.2
Concomitant surgery, *n* (%)	
Aortic valve replacement	19 (25.7)
Tricuspid valve surgery	15 (20.3)
Coronary artery bypass graft	2 (2.7)
Symptoms of heart failure, *n* (%)	50 (70.3)
NYHA I	3 (4.1)
NYHA II	28 (37.8)
NYHA III	13 (17.6)
NYHA IV	8 (10.8)
Hemoglobin (g/dL)	8.4 ± 1.8
LDH (IU/L)	1450 ± 1022
Total bilirubin (mg/dL)	3.3 ± 2.9
Transfusion requirement, *n* (%)	31 (41.9)
Creatinine (mg/dL)	1.08 ± 0.92
NT-pro BNP (pg/mL)	1465 ± 1799

Data presented as mean ± standard deviation or number and percentage. MV, mitral valve; NYHA, New York Heart Association; LDH, lactic dehydrogenase; NT-pro BNP, N-terminal pro B-type nitric peptide.

**Table 2:** Echocardiographic and cardiac computed tomography characteristics.

Variables	*n* = 74
Echocardiographic characteristics	
LV end-diastolic dimension, mm	52.6 ± 7.4
LV end-systolic dimension, mm	35.2 ± 6.3
LV ejection fraction (%)	64.2 ± 8.0
LA volume index, mL/m^2^	128.6 ± 99.1
MV MDPG, mmHg	6.4 ± 2.5
PVL severity, *n* (%)	
Mild	40 (54.1)
Moderate	24 (32.4)
Severe	10 (13.5)
MR velocity through the PVL, m/s	
Peak	5.4 ± 0.9
Mean	3.7 ± 0.7
SPAP, mmHg	55.2 ± 15.2
CT characteristics	
PVL hole area, cm^2^	0.83 ± 0.87
PVL perimeter, mm	41.3 ± 24.8
PVL location, *n* (%)	
Anteromedial	13 (17.6)
Posteromedial	15 (20.3)
Posterolateral	33 (44.6)
Anterolateral	5 (6.8)
Multiple PVL	4 (5.4)
LA calcification	
No, *n* (%)	20 (27.0)
Mild, *n* (%)	25 (33.8)
Moderate, *n* (%)	17 (23.0)
Severe, *n* (%)	10 (13.5)
LA calcium score	3507 ± 8057

Data presented as mean ± standard deviation or number and percentage. CT, computed tomography; LV, left ventricular; LA, left atrial; MV, mitral valve; MDPG, mean diastolic pressure gradient; PVL, paravalvular leakage; MR, mitral regurgitation; SPAP, systolic pulmonary artery pressure.

**Table 3:** Factors associated with hemolysis.

Hemolysis: LDH	Univariable			Multivariable *		
	β	*t*	*p* Value	β	*t*	*p* Value
Age, year	−0.086	−0.660	0.512			
Female sex	−0.041	−0.319	0.751			
Presence of atrial fibrillation	−0.081	−0.623	0.536			
LV ejection fraction, %	0.248	1.916	0.060	0.243	1.793	0.080
Previous mechanical MV	6.007	0.012	0.990			
Time to last MV surgery to diagnosis of PVL, year	−20.272	−1.284	0.204			
Multiple PVL	0.030	0.231	0.818			
PVL location (posterolateral)	0.003	0.025	0.980			
PVL hole area, cm^2^	0.036	0.262	0.794			
PVL perimeter, mm	−0.022	−0.158	0.875			
Peak MR velocity, m/s	0.271	1.912	0.062			
Mean MR velocity, m/s	0.345	2.495	0.016	0.345	2.495	0.016
Mean diastolic pressure gradient, mmHg	−9.320	−0.187	0.852			
LA volume index, ml/m^2^	−0.094	−0.709	0.482			
LA calcium score	−0.116	−0.899	0.372			

* Variables with a significance <0.1 in univariate analysis were entered into multivariate analysis. LV, left ventricular; PVL, paravalvular leakage; MR, mitral regurgitation; MV, mitral valve; MVR, mitral valve replacement; LA, left atrial.

**Table 4:** Factors associated with pulmonary hypertension.

Heart Failure: PASP	Univariable			Multivariable *		
	β	*t*	*p* Value	β	*t*	*p* Value
Age, year	0.017	0.142	0.888			
Female sex	0.122	1.001	0.321			
Presence of atrial fibrillation	0.124	1.006	0.318			
LV ejection fraction, %	0.136	1.106	0.273			
Previous mechanical MV	5.199	0.853	0.396			
Time to last MV surgery to diagnosis of PVL, year	0.071	0.305	0.761			
Multiple PVL	0.153	1.235	0.221			
PVL location (posterolateral)	−0.202	−1.675	0.099			
PVL hole area, cm^2^	0.272	2.156	0.035			
PVL perimeter, mm	0.277	2.199	0.032	0.249	2.151	0.036
Peak MR velocity, m/s	0.034	0.249	0.804			
Mean MR velocity, m/s	−0.066	−0.480	0.633			
Mean diastolic pressure gradient, mmHg	0.100	0.150	0.881			
LA volume index, ml/m^2^	−0.099	−0.800	0.427			
LA calcium score	0.364	3.147	0.002	0.467	3.992	<0.001

* Variables with a significance <0.1 in univariate analysis were entered into multivariate analysis. LV, left ventricular; PVL, paravalvular leakage, MR, mitral regurgitation; MV, mitral valve; LA, left atrial.

## Data Availability

The data presented in this study are available on request from the corresponding author. The data are not publicly available due to privacy.

## Supplementary Materials

The following are available online at https://www.mdpi.com/2077-0383/10/2/222/s1, Figure S1: (A) Patient with mitral PVL at a posteromedial location (Off-axis view) (B) Patient with mitral PVL at anteromedial location (Apical 3 chamber view), Figure S2: (A) PVL hole area (B) PVL perimeter according to the PVL severity classified by conventional guideline, Figure S3: Association between degree of hemolysis and PVL hole area, Figure S4: Associations between degree of hemolysis and MR velocity measured by TEE (A) mean MR velocity through the PVL (B) peak MR velocity through the PVL, Figure S5: Associations between mean mitral regurgitation (MR) velocity through the PVL and PVL hole area (A) and PVL perimeter (B).
