# Supplementary material for: Structural and Functional Characteristics of Mitral Paravalvular Leakage Identified by Multimodal Imaging and Their Implication on Clinical Presentation
Source: J Clin Med. 2021 Jan 10;10(2):222. doi: 10.3390/jcm10020222 (PMC7826927; doi:10.3390/jcm10020222)
Supplement: Supplementary file 1 [file jcm-10-00222-s001.pdf]

SUPPLEMENTARY DATA

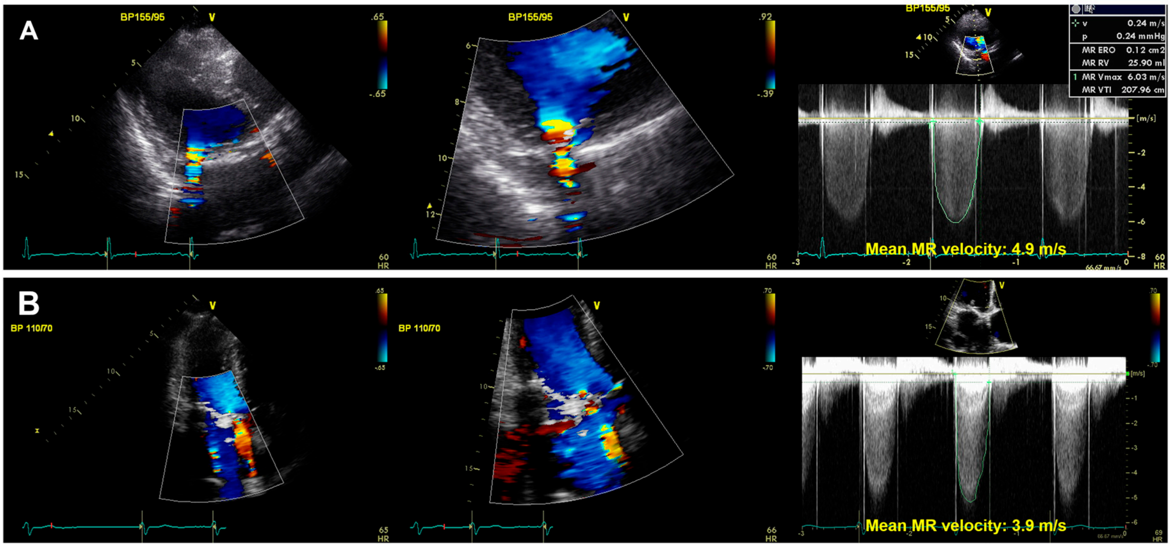

Figure S1. (**A**) Patient with mitral PVL at a posteromedial location (Off-axis view) (**B**) Patient with mitral PVL at anteromedial location (Apical 3 chamber view). PVL, paravalvular leakage.

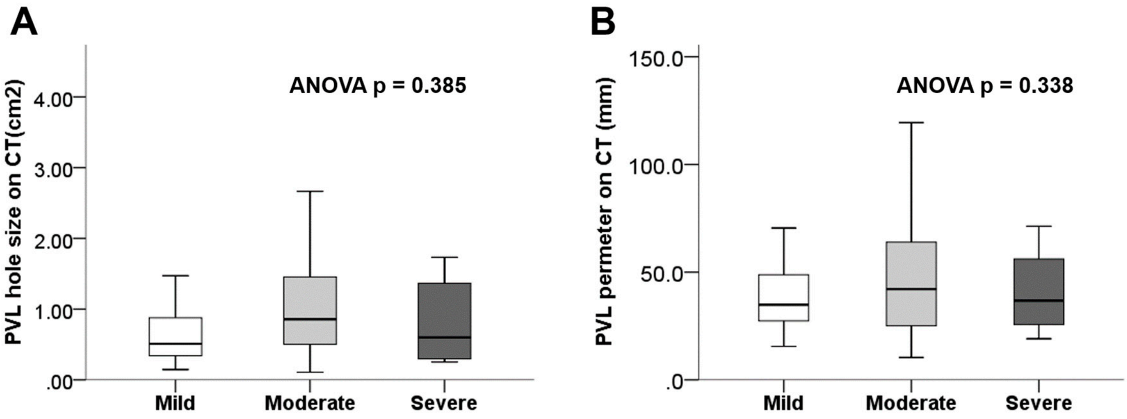

Figure S2. (**A**) PVL hole area (**B**) PVL perimeter according to the PVL severity classified by conventional guideline. PVL, paravalvular leakage.

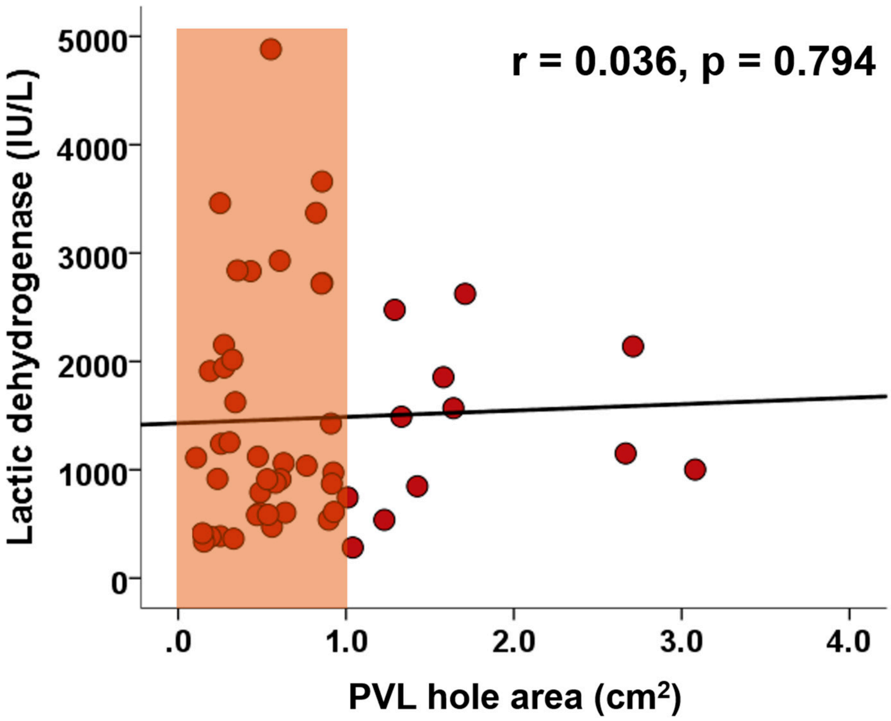

Figure S3. Association between degree of hemolysis and PVL hole area. PVL, paravalvular leakage.

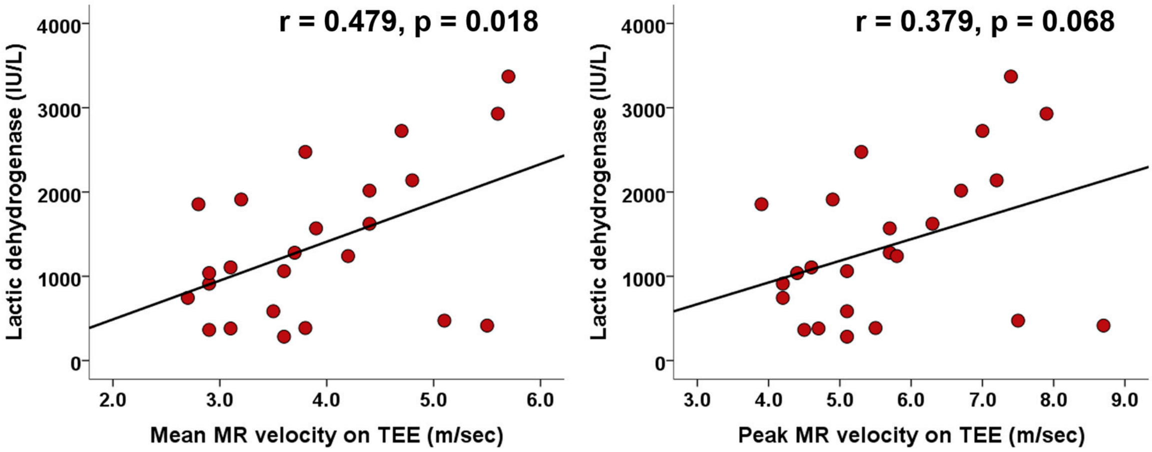

Figure S4. Associations between degree of hemolysis and MR velocity measured by TEE (**A**) mean MR velocity through the PVL (**B**) peak MR velocity through the PVL. MR, mitral regurgitation; PVL, paravalvular leakage.

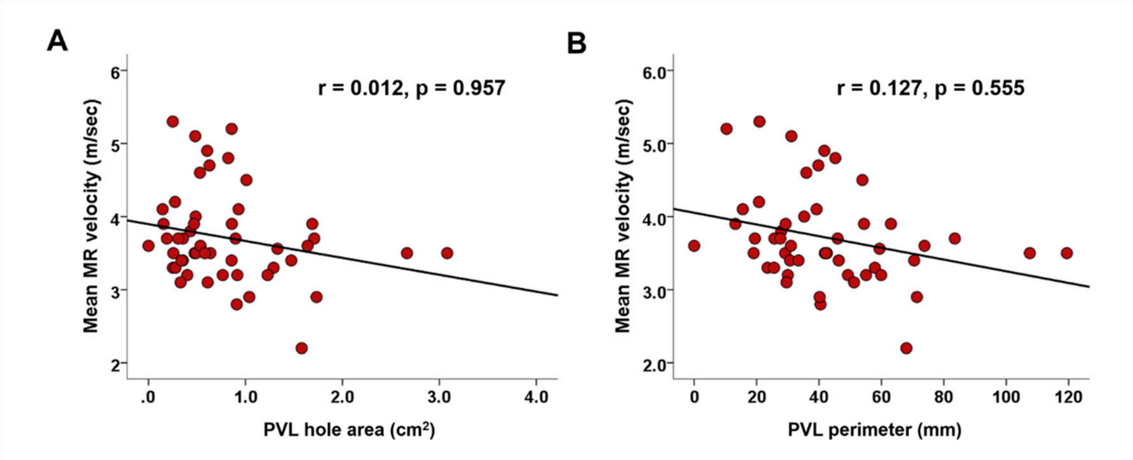

Figure S5. Associations between mean MR velocity through the PVL and PVL hole area (**A**) and PVL perimeter (**B**). MR, mitral regurgitation; PVL, paravalvular leakage
